# Who cares about the carers? A call to action on behalf of mothers of disabled children

**DOI:** 10.3389/fsoc.2023.1304676

**Published:** 2024-01-11

**Authors:** Alison Pearson

**Affiliations:** Department of Health and Community Sciences, University of Exeter, Exeter, United Kingdom

**Keywords:** disabled children, maternal wellbeing, parent carers, COVID-19, employment, pandemic

## Abstract

This ‘perspective’ article is an urgent call to action on behalf of mothers (and indeed fathers) of disabled children in the wake of the COVID-19 pandemic. In doing so draws attention to the ‘perfect storm’ experienced by United Kingdom-based parents of reduced support and increased stress, coupled with the effects of isolation. It also illuminates some of the impact of actions taken, and not taken, during and after the pandemic and the enduring effects for working parent-carers. In doing so I argue that, despite facing these extreme challenges, these parents have been a neglected group from both a policy and research perspective. The call to action therefore extends to society, employers, and to the research community.

## Why a call for action is needed

The wellbeing of some of the most vulnerable in society—that of disabled children—is largely dependent on the ability of their parents to care for them, and to advocate for support and services on their behalf. Employment may be a financial necessity, but also in itself contributes to wellbeing and participation within society (Prainsack and Buyx, 2018). The environment and support provided therefore enables the whole family to survive and thrive, or not. This has, however, been a neglected area from both a policy and research perspective: both for parent-carers as a whole and for mothers in particular. This paper is therefore an urgent call to action in the wake of the COVID-19 pandemic, which has both illuminated the issues and exacerbated the problems.

The challenges of parenting a disabled child include significant levels of practical and emotional work, with high levels of exhaustion reported as the result of often round-the-clock care (Doig et al., 2009). Parents must rapidly become experts in a wide range of other professional therapeutic activities for their child: for example, nursing, physiotherapy, and specialist feeding. As well as the practicalities of care, parents also face extra tasks such as managing medical appointments and additional administrative work (Caicedo, 2014). This impact is not just physical, but also emotional: parents report having to take on an adversarial role with services that are supposed to help them (Fazil et al., 2002). There is further emotional stress of coping with the after-effects of a diagnosis, in turn exacerbated if the child has a serious health condition (Caicedo, 2014). Despite these acknowledged challenges, reports consistently indicate a shortage of support services and significant levels of underfunding (O’Hagan and Kingdom, 2020). For example, a United Kingdom survey identified that only 4% of parents felt they were receiving the support they needed (Disabled Children’s Partnership, 2019). It is perhaps unsurprising, therefore, that parent-carers are recognised as being at much higher risk of both physical and mental ill-health compared to other parents (Gallagher et al., 2008; Bjornstad et al., 2021).

It has been widely acknowledged that childcare during the COVID-19 pandemic fell disproportionately to mothers (O’Reilly, 2020; Collins et al., 2021; Zamarro and Prados, 2021). For most families, who were not previously home-schooling, school closures and the introduction of online learning created significant challenges for mothers’ work and wellbeing (Petts et al., 2021; Khan, 2022). Whilst the balance of care between mothers and fathers of disabled children during the pandemic is under-researched, older research suggests that women are more likely to be the primary caregiver for disabled children (Rogers and Hogan, 2003; Findler et al., 2016). It is therefore likely that wellbeing and workplace participation is particularly affected for mothers: however, this paper also highlights challenges faced by *both* parents of children with disabilities. As such ‘parent-carers’ will be referred to, recognising the disadvantage faced by this whole set of parents, of whom it is anticipated (but not *known*) that mothers are most impacted of all. An essential element of the call for action is therefore to the research community: to investigate the different issues faced by mothers and fathers, and the intersection with work and wellbeing.

The challenges of caring for a disabled child—before, during and after the COVID-19 pandemic—are influenced by many locality-specific factors including health and social care systems, government policy, legislation, employment opportunities, financial support and resources. Within this article, the United Kingdom situation is used as an example to illustrate some of the points made, and to highlight the urgency of this call to action.

## The effects of the pandemic on mothers of disabled children

### The situation before the pandemic

To appreciate the impact of the pandemic, it is valuable to consider the pre-pandemic context. It had already been reported that parent-carers are more likely to experience discrimination, marginalisation and economic disadvantage (Fazil et al., 2002). It is perhaps worth noting that much of the data has been collected and reported by charities rather than through government-commissioned research, even though 11% of United Kingdom children have been identified as having a disability (Department for Work and Pensions, 2023).

Where United Kingdom parent-carers are employed, there are no specific nationally-directed policies to provide additional support. For example, ‘the right to request flexible working for parents and carers of disabled children is the same as parents of non-disabled children’ (Working Families, 2022). Parents of disabled children can take time off for parental leave, in line with other parents, but not to be paid for this (UK Government, 2023), regardless of their child’s health or disability status. There are no government-based employment policies to give additional or tailored support to parents of disabled children, and employers may be unaware of how many parent-carers are within their organisation, given that they are not required to collect this data (Morton-Young, 2021). The impact on parents is significant: for example, a survey by Working Families (2015) found that 76% had taken a demotion or not applied for a promotion because of their caring responsibilities, with significant numbers unable to find suitable employment at all. Similarly, Caicedo (2014) reported that a third of parents had stopped work altogether at some point to care for their child full-time. This context is important when considering the pandemic’s impact, as the challenge of work for parent-carers existed well before the events of 2020.

### The impact of the pandemic

Given the sudden arrival of the pandemic, it is no surprise that parent-carers experienced difficulties. However, even as early as three weeks into the first school closures, some of these parents were reporting feeling ‘at breaking point’ (O’Hagan and Kingdom, 2020, p. 230), with significant majorities in repeated surveys indicating considerable stress and distress (Disabled Children’s Partnership, 2020, 2021a,b). These parents were experiencing a compounding set of experiences: the loss of the structure and therapies provided by school or other providers, the physical and mental pressures of now caring for their children full time, and the loss of respite care. Furthermore, many disabled children were identified as being ‘clinically extremely vulnerable’ and at much higher risk of serious outcomes from COVID-19. As well as additional stress caused by this identification, such families were advised to take part in some form of ‘shielding’, with government advice in March 2020 including ‘staying at home at all times and avoiding any face-to-face contact until at least the end of June’ (UK Government, 2020, p. 2). Further stress was added through concerns about child development—or even regression—when therapies ceased (Merrick et al., 2023), in increased challenging behaviours for some families (Wolstencroft et al., 2021), and negative impacts on their child’s mental health (Banerjee et al., 2021). Families were therefore caught in a ‘perfect storm’ of reduced care, increased stress, and isolation from others who may have been able to help, physically or emotionally. At the same time, many disabled children were unable to continue any level of learning through online methods or needed considerable parental input to do so (Bakaniene et al., 2023), giving parents more responsibility for their education and development. Given these children’s additional needs, this education is often considerably more challenging and requires high levels of expertise, adding further difficulties for parents.

There was also added emotional stress. In a paper titled ‘Your only is my everything’ Pozniak and de Camargo (2021) foreground the challenges for parents when daily media reports highlighted that ‘only’ those with underlying health conditions were dying from COVID-19. Not only were these parents facing additional caring responsibilities through the closure of school and support services, but they were emotionally isolated by suggestions that their children did not ‘count’ as much as a ‘healthy’ child. It could easily be suggested that these prejudices against the disabled were already embedded within societal inequalities: nonetheless it had previously been rare to see them articulated quite so starkly.

Whilst the additional strain experienced by parent-carers was identified relatively early in the pandemic (Dhiman et al., 2020; O’Hagan and Kingdom, 2020), within the United Kingdom, no targeted action was taken to support either disabled children or their parents: indeed no such support was implemented at any stage of the pandemic, and has not featured in any post-pandemic recovery plans. For example, whilst plans were introduced to support the learning lost by children in general (UK Government, 2021a,b), none of the £700 million announced was targeted towards disabled children. No additional funding for disabled children or their families has been announced, even in 2023, despite reports that children with special educational needs and disabilities (SEND) have experienced delays in provision and higher absence levels (Ofsted, 2022). Given that significant stress reported by parents during the pandemic was due to their children not making educational or developmental progress (Merrick et al., 2023), the lack of government acknowledgement of this problem means that it is hard to be optimistic about this situation improving.

Given the sustained level of emotional and physical challenges, and that COVID-19 continues to present a risk to those with medical vulnerabilities, it is perhaps unsurprising that parent-carers continue to experience the effects of pandemic-related stress (Disabled Children’s Partnership, 2022): their needs once again diverging from the ‘return to normal’ experienced by many in the wider population.

### Juggling employment and caring during the pandemic

As with any parents, parent-carers of disabled children may need (or choose) to work for several reasons. Even without the current cost of living crisis, having a disabled child is likely to result in additional costs which are not covered by government benefits (Fazil et al., 2002). In many countries, there is limited or no additional financial support for parent-carers, creating an economic imperative for many to work. Parents may also need work from an emotional or intellectual perspective: employment has been extensively linked with mental health benefits (Zabkiewicz, 2010), including improved social connections, a sense of purpose and self-worth (Modini et al., 2016). Likewise, unemployment has been associated with increased risks of mental ill-health and lower psychological wellbeing (McKee-Ryan et al., 2005).

The onset of the pandemic was undoubtedly challenging for very many, particularly those mothers trying to juggle childcare, home learning, their own employment and running a household. Working parents of disabled children experienced an extreme version of this challenge, with many of these children unable to learn independently, or even to be left unsupervised in another room, and many needing full time care: none of which is congruent with parents continuing with any semblance of normal working. Given the disproportionate role of mothers in child-caring (Rogers and Hogan, 2003), it is likely that this effect was felt most keenly amongst working mothers. Whilst United Kingdom children with Education, Health and Care Plans were allowed to continue to attend school for some of the national lockdown periods (Department for Education, 2020), in practice many remained at home following government advice for the vulnerable to avoid all face-to-face contact (O’Hagan and Kingdom, 2020).

### The legacy of the pandemic

For a brief moment, some parent-carers reported that the rest of the world had an insight into some of the challenges and isolation that they faced in their everyday lives, providing a glimmer of hope that their needs might be better recognised in future (Pozniak and de Camargo, 2021).

However, rather than using the lessons of the pandemic to strengthen the support for these parents, recent reports have identified that parent-carers are struggling more than ever (Disabled Children’s Partnership, 2022). There is no doubt that the pandemic, and those actions taken and not taken, have both illuminated and amplified existing disadvantage gaps: for example those of gender (Zamberlan et al., 2021), race (Haynes, 2020) and socio-economic status (Blundell et al., 2022). It is heartening to see that there has been considerable concern about the disproportionate effect of the pandemic on mothers. The gap between mothers (or fathers) of disabled children and other mothers (or the rest of the population), however, has received considerably less attention, either before, during or after the onset of the pandemic. Without sufficient data collection, or even acknowledgement of this gap, it is left to interested charities to research. The results are stark. A recent survey of 2,200 parent-carers reported than since the start of the pandemic 75% have had to cut back on or give up work completely, half are now living in poverty, and 87% reported a negative impact on their mental health (Disabled Children’s Partnership, 2022). The challenge is exacerbated by the impact on the pandemic on health and social care: many disabled children are also now facing increased delays and reduced access to support services (Disabled Children’s Partnership, 2022).

As well as demographic characteristics, the lens of psychological resources has also been used to examine the differential effects of the pandemic. For example, Alat et al. (2023) identified that psychological capital, together with an internal locus of control, may have had a strong mediating effect in reducing the negative mental health impacts of periods of lockdown. The value of positive psychological approaches in mediating against pandemic-related stress has been reported both early in the pandemic (for example, Hagger et al., 2020) and more recently (Kraus et al., 2023). However, these studies have largely focussed on the general population or sections of the workforce: given that many parent-carers were already experiencing sustained psychologically challenging situations before the pandemic, the protective value (or not) of their psychological capital might also warrant particular attention.

## Discussion

In many ways, the pandemic was just the call to action that the parent-carers should have benefited from. For the first time, in living memory, there were national and international debates about the importance of both social interactions and work, the detrimental effects on mental health and wellbeing when these are missing, the impact of health concerns and the effects of social isolation. The arguments that parent-carers had been making as individuals behind the scenes were writ large across a world stage. Yet, rather than building on the adaptations to work, commuting and connectivity necessitated by the pandemic, the gap between the now ‘unworried well’ and those with disabilities and health conditions has increased rather than diminished.

In considering instead the lessons which *could* be learned, this paper is a call for action: at a societal level, to employers, and in future research.

### For society

In a socially-just world there would be a moral imperative to take action, but even without this, the financial and health resources argument would be to support parents to remain well and able to care for their children. The COVID-19 pandemic may well have illuminated existing problems and disadvantage experienced by parent-carers (O’Hagan and Kingdom, 2020), but articles written early in the pandemic were at least hopeful that the recognition of the impact of isolation would lead to a permanence in newly-established changes: unfortunately these hopes have not been realised. The creation of ‘certain accommodations from which these families are poised to benefit’ (Pozniak and de Camargo, 2021, p. 280) has, in fact, turned out to be temporary now that the able-bodied can return to their pre-pandemic ‘normal.’

Moving forward, the choices are stark. If societies fail to support parent-carers, then the risk is that either the needs of disabled children will not be met, or that this falls completely to a health and social care sector already on the brink of collapse. Likewise both the physical and mental health of parent-carers is in jeopardy, with studies reporting increased depression and hopelessness (Figueiredo et al., 2020), and a much higher level of suicidal thoughts (O’Dwyer et al., 2023) than the general population.

There are no easy answers, but this situation is unlikely to improve unless at least acknowledged, and the needs of parent-carers included in the scoping of post-pandemic health and social care support and services. The response needed is likely to be complex, and implemented at multiple levels. These parents were already experiencing difficulties, and over the last three years have faced significant challenges to their health and wellbeing: one-size-fits all solutions are unlikely to be successful given their diverse situations and needs (Griffin and Gore, 2023).

### For employers

In considering the role of employers in supporting parent-carers, it is all too easy to consider that the argument for doing so is purely one of fairness. However, even where employers are disinterested in social justice, they may consider benefits from both a utilitarian and reputational perspective: not just how employment benefits the individual, but how the individual may benefit the employment. This parent group is one that is likely to understand trauma, complexity and managing multiple priorities: their experiences may have also helped them to gain greater emotional awareness (Griffin and Gore, 2023). They have frequently had to advocate on behalf of disadvantage, to manage many simultaneous competing demands, and to demonstrate extreme levels of perseverance in the face of adversity. Perhaps, as we move through the 21st century, we might celebrate the skills and qualities that parent-carers almost inevitably fulfil in their outside-work lives and recognise these within the workplace. The argument has been well rehearsed that disability should be seen as a social rather than a medical model (Bunbury, 2019): maybe this should be extended to parent-carers too, recognising that they have positive qualities and skills and need support, rather than being seen through the deficit-model perspective as people who simply need extra time off or more flexibility. As employers have experienced major recruitment and retention difficulties, particularly in people-based public services (Office for National Statistics, 2023), now might be the time to consider the positive value of parent-carers to the workplace. A first step for employers might be to at least identify how many parent-carers are within an organisation, and then to ascertain how they might be supported to ensure better equity, in the same way as is expected for other disadvantaged groups within equality, diversity and inclusion strategies.

### For research

This is also a call to action for further research. There has been limited policy consideration or research investigating the impact of COVID-19 on disabled people at all (Shakespeare et al., 2022; Read et al., 2023). As a subset of this population, the research including disabled children has been even more limited, and the enduring effects on their mothers (or fathers) even less so. Given the paucity of research into the specific challenges faced by working parents of disabled children, this is a critical step in gaining the understanding needed to develop more supportive employment practises.

Whilst the mental health and wellbeing of the general population during the COVID-19 pandemic has been well reported (Penninx et al., 2022), there are rather fewer reporting on maternal (or paternal) wellbeing for parents of disabled children. Even recently, O’Connor Bones et al. (2022) identified a dearth of research investigating the effects of school closures on parents with children attending special schools. Even then, this is not a homogenous group, in terms of the nature of the disability, the parental situation and the impact of both of these on health and education. For example, even within a comprehensive scoping review reporting the challenges of online learning for pupils with additional needs (Bakaniene et al., 2023), the research did not include children with complex or profound disabilities who were unable to utilise online learning platforms at all.

Given the significant impacts noted in the few studies which have investigated the effect on parents (Disabled Children’s Partnership, 2022), it is important that the ongoing impact for parent-carers is investigated more extensively, including the impact on their employment and which employment practises are most helpful. Research is also urgently needed which recognises parent-carers are not a homogenous group. The limited evidence available suggests that mothers are more likely to have the greatest share of caring responsibilities for disabled children (Rogers and Hogan, 2003) and therefore are likely to suffer the greatest disadvantage, but further research is needed investigating the intersection of parent-carer experiences with other parental characteristics (gender, race, single parenting, parental disability, economic status etc.). It would also be helpful to investigate the experiences of caring for children with different types of disability: the experiences of parents may be very different, for example, in caring for a child with autism, physical mobility issues or complex learning and developmental delays. The specific examples reported in this paper have had a United Kingdom focus. It is also therefore important to recognise that differences in other countries in terms of government, infrastructure, policy and approaches to disability will affect the nature of both challenges and support experienced.

Significant work in research and policy will be needed to both support parent-carer mental health and wellbeing, and to enable the active participation and valuing of mothers (and fathers) of disabled children in the workplace. Caring about the carers has now assumed an urgency, both illuminated and exacerbated by the COVID-19 pandemic: it is a call that needs to be heeded, both for the mental and physical health and wellbeing of these parents and their children, and for the sake of society overall.

## Data availability statement

The original contributions presented in the study are included in the article/supplementary material, further inquiries can be directed to the corresponding author.

## Author contributions

AP: Conceptualization, Writing – original draft, Writing – review & editing.
